# Early acute kidney injury in nonsepsis, noncardiac surgical patients admitted to a general surgical ICU

**DOI:** 10.1186/cc13559

**Published:** 2014-03-17

**Authors:** KS Kongsayreepong, N Rojanapithayakorn

**Affiliations:** 1 Siriraj Hospital, Mahidol University, Bangkok, Thailand

## Introduction

Perioperative AKI is a significant factor determining morbidity/mortality in surgical patients. We sought to determine the prevalence and risk factors of early AKI (within 72 hours of ICU admission) in nonsepsis, noncardiac surgical patients admitted to the general surgical ICU.

## Methods

This prospective observational study was done in 600 nonsepsis, noncardiac surgical patients admitted to the 14-bed general surgical ICU of Siriraj Hospital. The following data were collected: patient demographic data, ASA, comorbidity, type and urgency of surgery, type of anesthesia, preoperative and the first 72 hours laboratory data, amount of bleeding, type/amount of fluid and blood replacement, average intraoperative and the first 72 hours MAP, and severity score. Outcome as ventilator-hours, ICU length of stay and ICU mortality were also determined. Risk factors were identified by multiple logistic regression. AKI was defined and classified according to the AKIN criteria using adjusted serum creatinine (Cr) [1].

## Results

In total, 41.7% of the study patients developed AKI (AKIN-I 31.0%, AKIN-II 10%, AKIN-III 4.8%) and 4.8% received RRT. The following factors were different between AKI and non-AKI patients: baseline expected GFR <60 ml/minute/1.73 m^2^, baseline serum Cr and baseline serum albumin, major abdominal surgery, vascular surgery, combined regional and general anesthesia, APACHE II score, receiving 6% 130/0.4 hydroxyethyl starch (HES) >20 ml/kg/day, 4% gelatin >20 ml/kg/day, crystalloid >30 ml/kg/day and positive fluid balance in the first 48 hours. Multiple logistic regression showed that independent risk factors of AKI included: baseline eGFR <60 ml/minute/1.73 m^2 ^(OR = 1.53; 95% CI, 1.08 to 1.27, *P *= 0.02), baseline serum albumin <2 mg/dl (OR = 1.75; 95% CI, 1.01 to 3.06, *P *= 0.049), admitting hemoglobin <8 g/dl (OR = 2.41; 95% CI, 1.14 to 5.11, *P *= 0.02), receiving 6% 130/0.4 HES >20 ml/kg/day (OR = 2.02; 95% CI, 1.09 to 3.76, *P *= 0.03), receiving crystalloid >30 ml/ kg/day (OR = 3.15; 95% CI, 1.53 to 6.48, *P *= 0.01), vascular surgery (OR = 1.67; 95% CI, 1.05 to 2.64, *P *= 0.03), and major abdominal surgery (OR = 1.68; 95% CI, 1.11 to 2.55, *P *= 0.01). AKI patients had higher ventilator- hours (*P *= 0.02) and ICU length of stay (*P *= 0.04).

## Conclusion

The prevalence of early AKI in nonsepsis, noncardiac patients admitted to the general surgical ICU was 41.7%. Surgical patients with risk factors should be carefully cared for to prevent the development of AKI. Receiving 6% 130/0.4 HES >20 ml/kg/day and crystalloid >30 ml/kg/day increased the risk of early AKI in nonsepsis, noncardiac surgical patients.
